# Modulation of SERCA in Patients with Persistent Atrial Fibrillation Treated by Epicardial Thoracoscopic Ablation: The CAMAF Study

**DOI:** 10.3390/jcm9020544

**Published:** 2020-02-17

**Authors:** Celestino Sardu, Gaetano Santulli, Germano Guerra, Maria Consiglia Trotta, Matteo Santamaria, Cosimo Sacra, Nicola Testa, Valentino Ducceschi, Gianluca Gatta, Michele D’ Amico, Ferdinando Carlo Sasso, Giuseppe Paolisso, Raffaele Marfella

**Affiliations:** 1 Department of Advanced Medical and Surgical Sciences, University of Campania “Luigi Vanvitelli”, 80138 Naples, Italy; mariaconsiglia.trotta@unicampania.it (M.C.T.); gianluca.gatta@unicampania.it (G.G.); ferdinandocarlo.sasso@unicampania.it (F.C.S.); giuseppe.paolisso@unicampania.it (G.P.); raffaele.marfella@unicampania.it (R.M.); 2 Department of Medical Sciences, International University of Health and Medical Sciences “Saint Camillus”, 00131Rome, Italy; 3 Department of Advanced Biomedical Sciences, International Translational Research and Medical Education Academic Research Unit (ITME), “Federico II” University, 80138 Naples, Italy; gsantulli001@gmail.com; 4 Department of Medicine, Albert Einstein College of Medicine, Fleischer Institute for Diabetes and Metabolism (FIDAM), Montefiore University Hospital, New York, NY 10461, USA; 5 Department of Medicine and Health Sciences "Vincenzo Tiberio", University of Molise, 86010, Campobasso, Italy; germano.guerra@unimol.it; 6 Department of Experimental Medicine, University of Campania “Luigi Vanvitelli”, 80138 Naples, Italy; matteo.santamaria@gemellimolise.it (M.S.); micheledamico@unicampania.it (M.D.A.); 7 Cardiovascular and Arrhythmias Department, Catholic University of Sacred Heart, 86010 Campobasso, Italy; cosimo.sacra@gemellimolise.it (C.S.); nicola.testa@gemellimolise.it (N.T.); 8 Cardiovascular and Arrhythmias Department, “Vecchio Pellegrini” Hospital; 80138 Naples, Italy; valentino.ducceschi@tin.it

**Keywords:** persistent atrial fibrillation, epicardial ablation, calcium channels, SERCA

## Abstract

Objectives: To evaluate atrial fibrillation (AF) recurrence and Sarcoplasmic Endoplasmic Reticulum Calcium ATPase (SERCA) levels in patients treated by epicardial thoracoscopic ablation for persistent AF. Background: Reduced levels of SERCA have been reported in the peripheral blood cells of patients with AF. We hypothesize that SERCA levels can predict the response to epicardial ablation. Methods: We designed a prospective, multicenter, observational study to recruit, from October 2014 to June 2016, patients with persistent AF receiving an epicardial thoracoscopic pulmonary vein isolation. Results: We enrolled 27 patients. Responders (n = 15) did not present AF recurrence after epicardial ablation at one-year follow-up; these patients displayed a marked remodeling of the left atrium, with a significant reduction of inflammatory cytokines, B type natriuretic peptide (BNP), and increased levels of SERCA compared to baseline and to nonresponders (*p* < 0.05). Furthermore, mean AF duration (Heart rate (HR) 1.235 (1.037–1.471), *p* < 0.05), Left atrium volume (LAV) (HR 1.755 (1.126–2.738), *p* < 0.05), BNP (HR 1.945 (1.895–1.999), *p* < 0.05), and SERCA (HR 1.763 (1.167–2.663), *p* < 0.05) were predictive of AF recurrence. Conclusions: Our data indicate for the first time that baseline values of SERCA in patients with persistent AF might be predictive of failure to epicardial ablative approach. Intriguingly, epicardial ablation was associated with increased levels of SERCA in responders. Therefore, SERCA might be an innovative therapeutic target to improve the response to epicardial ablative treatments.

## 1. Introduction

Atrial fibrillation (AF) is the most common arrhythmia worldwide [1]. Based on the duration of episodes and the date of onset, AF is defined as paroxysmal, persistent, or permanent [1]. In patients with persistent AF there is an increased risk of thromboembolic stroke, heart failure, and overall worse prognosis [2]. Therefore, in these patients catheter ablation is a valid therapeutic option to ameliorate clinical outcomes via restoration of sinus rhythm [2,3,4]. On the other hand, catheter ablation shows a success rate of ~50% at five years in patients with persistent AF [5]. The lack of therapeutic effect in a relatively high percentage of patients limits its clinical application, and might itself be responsible for atrial fibrosis and remodeling, eventually leading to permanent AF [6,7]. Unfortunately, in these patients the successful epicardial ablation by sinus rhythm restoration could cause a reduction of left atrial diameters and volumes [6,7,8,9]. In this setting, epicardial AF ablation could induce the modulation of the complex electro-anatomical arrhythmic atrial substrate in patients with persistent AF [6]. Conversely, the unsuccessful of AF ablation could be explained by the advanced atrial electrical/anatomical remodeling [6,7,8,9], as the result of an enhanced trigger activity and reentry mechanisms [10]. These mechanisms are both implied in the genesis and perpetuation of persistent AF [11]. Alterations in the regulation of intracellular calcium (Ca^2+^) have been linked to an abnormal trigger activity and reentry in AF patients [10,12]. Moreover, in human atrial myocytes altered Ca^2+^ fluxes have been shown to induce delayed after depolarizations (DADs) [9,10,11,12]. Sarcoplasmic Endoplasmic Reticulum Ca^2+^ ATPase (SERCA) is considered a major player in these processes [10]. Indeed, patients with AF have lower SERCA levels compared to patients with sinus rhythm [13]. Of note, SERCA levels can be assayed in peripheral blood lymphocytes, as their levels correlate with SERCA levels obtained in specimens of cardiac tissue [13]. Mechanistically, a reduced Ca^2+^ uptake in the endoplasmatic/sarcoplasmatic reticulum, which is mediated by SERCA, results in intra-cytoplasmic Ca^2+^ overload, which is known to be arrhythmogenic [10,14]. Moreover, the abnormal Ca^2+^ handling along with cellular DAD-mediated triggered activity could promote AF persistence [10,11,12,13,14,15]. Indeed, the persistence of abnormal Ca^2+^ handling can activate ion channels and trigger Ca^2+^-dependent signaling pathways, eventually promoting atrial remodeling and the progression of AF to more persistent forms [10]. Therefore, we speculate that alterations in SERCA levels might play a central role in AF persistence and in its recurrence following an epicardial ablation. To our knowledge, these aspects have never been investigated before. Our hypothesis is that lower SERCA levels might be linked to higher rates of failure of epicardial ablation in patients with persistent AF. To verify such hypothesis, we designed a prospective, multicenter, observational study to evaluate AF recurrences at one year of follow-up after epicardial ablation, correlating this clinical outcome to SERCA expression in patients with sinus rhythm restoration (responders group) vs. patients with AF (nonresponders group) after an epicardial ablative approach.

## 2. Methods

### 2.1. Study Design

From October 2014 to June 2016, we recruited consecutive patients with persistent, symptomatic AF, and for ≥6 months refractory to ≥1 class 1–3 antiarrhythmic drugs (AADs) in the prospective, multicenter, observational study CAMAF (Ca^2+^ ATPase Modulates Atrial Fibrillation) at “Vecchio Pellegrini” Hospital, Naples, Italy, at University of Naples “Federico II”, Naples, Italy, at University of Campania “Luigi Vanvitelli”, Naples, Italy, at Catholic University of Sacred Heart, Campobasso, Italy, and at University of Molise, Campobasso, Italy. Exclusion criteria were: Patients aged <18 or >75 years, with structural heart diseases, left ventricle ejection fraction <30%, and left atrium diameter >55 mm; patients affected by any condition that would make survival for one year unlikely; and patients with prior cardiac surgery. All enrolled patients received an epicardial thoracoscopic (closed heart) video assisted pulmonary vein isolation (PVI). AF was defined according to the international guidelines for the management of patients with AF [2]. Persistent AF was defined as continuous AF lasting longer than 7 days [2]. Before interventions and at follow-up, baseline laboratory studies, including glycated hemoglobin A1c (HbA1c), lipid panel, fibrinogen, C reactive protein (CRP), interleukin 6 (IL-6), tumor necrosis factor alpha (TNFα), B type natriuretic peptide (BNP), and SERCA levels were evaluated. These biomarkers were evaluated in the overall study population, and at one-year follow-up in responders vs. nonresponders. The Institutional Committees on Human Research at Participants Centers approved the study (Approval number 1022014). The study was performed according to the declaration of Helsinki. All patients were informed of the nature of the study and provided written consent.

### 2.2. Anthropometric Evaluation

For all patients, we performed a full clinical assessment that included physical examination, vital signs, and the review of adverse events. In addition, at every visit we performed the fasting blood (at least 12 h from last meal) for glycemia, lipid profile (total cholesterol, triglycerides, and high-density and low-density cholesterol), and C reactive protein (CRP).

### 2.3. Pre-Operative Management

All patients with persistent AF were under oral anticoagulation therapy (vitamin K antagonists, VKA, or new oral anticoagulation drugs). Three to five days before the epicardial ablation procedure we discontinued all oral anticoagulation drugs, replaced by subcutaneous fractionated heparin. To rule out the presence of atrial thrombi, all patients received a trans-esophageal echocardiography within 48 h before the epicardial ablation procedure. AADs were discontinued for three half-lives before the ablative treatment.

### 2.4. Epicardial Catheter Ablation Procedure

The goal of the procedure is the electrical isolation of pulmonary veins and posterior left atrium wall by creating a continuous circumferential ablation line around all four pulmonary veins (‘‘box lesion’’) using a fully thoracoscopic, unilateral, off-pump approach. The procedure was conducted in general anesthesia and with selective left lung ventilation, using a direct optics video-thoracoscopic camera, with a right thorax access by three working ports, as described [6]. We opened the pericardium anterior to a phrenic nerve, and we dissected the transverse and oblique sinuses; then, we inserted the ablation catheter. Before the ablation, the correct position of the catheter was confirmed by trans-esophageal echocardiography [6]. We used the COBRA Fusion TM 150 (Estech, San Ramon, CA, USA) ablation system with the use of temperature-controlled, both monopolar and bipolar, RF energy, and with a suction design that eliminated the heat sink effect. We performed a circumferential movement of the catheter between the ablation cycles to close the box lesion, with a temperature setting of 70 **°**C and performing two cycles of 60 s each. Thereafter, we performed a direct current cardioversion for patients who were not in sinus rhythm after ablation. Subsequently, we introduced a tetrapolar catheter (Josephson curve catheter, Webster, Diamond Bar, CA USA) via the internal right femoral vein access to test a conduction block by pacing right atrium and right pulmonary veins [2]. This tetrapolar catheter was advanced first in the right atrium, and then positioned within the box lesion. Then, via the right internal jugular vein we advanced into the right atrium a deflectable curve catheter (Webster, Diamond Bar, CA USA) that was positioned in the coronary sinus to confirm potential captures (exit block). The procedure was concluded after the confirmation of a bidirectional (entrance and exit) block. In absence of complications and with stable hemodynamic conditions, treated patients were transferred to the intensive care unit.

### 2.5. Peri-Operative and Post-Operative Care

We removed chest drains after a chest radiography. After evaluation of bleeding risk, patients were given unfractionated heparin, continued until international normalized ratio was >2.0. Oral anticoagulants and AADs were continued for all blanking period after procedure. The blanking period was between 72 h and 3 months post-ablation [2]. In case of AF recurrences, episodes lasting 24 h were treated with an electric cardioversion. After clinical and instrumental assessment, patients were discharged and followed to outpatient clinic.

### 2.6. Blood Sampling

We collected blood samples from peripheral vein accesses at baseline (before epicardial ablation), and at 12-month follow-up after epicardial ablation.

### 2.7. Cytokines

Peripheral venous blood was drawn after an overnight fast, at breakfast time. We stored serum samples at −80 °C, and we measured the serum concentrations of TNFα and IL-6 in duplicate ways by using a highly sensitive quantitative sandwich enzyme assay (ELISA, Quantikine HS; R&D Systems, Minneapolis, USA).

### 2.8. Isolation of Human Lymphocytes

We isolated circulating lymphocytes from venous blood and we assayed SERCA expression, as previously reported [16,17]. Briefly, 3 mL of whole blood were added to a density gradient media (1114683 PolymorphPrep™, Progen, Germany) and centrifuged at 500×g for 30 min at 20 °C. After centrifugation, the layer containing peripheral blood mononuclear cells was transferred to a fresh tube by using a P1000 pipette, washed twice with phosphate buffer saline, centrifuged at 500×g for 5 min each time, and transferred to a T-75 culture flask in 20 mL RPMI 1640 media (10% FBS and 1% penicillin/streptomycin). Cells were then incubated at 37 °C and 5% CO_2_ for 1 hour, in order to separate the monocytes adherent to the flask surface from the lymphocytes remaining in suspension. The cell medium containing lymphocytes was removed from the flask and centrifuged at 500×g for 5 min. The pellet was re-suspended and trypan blue dye was added to determine the number of viable lymphocytes present in the suspension.

### 2.9. Protein Isolation and ELISA Test

Following lymphocytes’ lysis in RIPA buffer (R0278 Merck, Darmstadt, Germany) containing a complete protease inhibitor and phosphatase cocktail (11873580001 Roche, Basel, Switzerland), cell lysates were centrifuged at 12,000 rpm for 10 min at 4 °C [16]. Protein concentrations of supernatants were quantified with Bio-Rad protein assay (500-0006 Bio-Rad Laboratories, Hercules, CA) in order to assess SERCA2 protein levels by ELISA assay (Human SERCA2 Elisa kit, LS-F6830 LifeSpan Biosciences, Seattle, WA) following the manufacturer’s instructions. 

### 2.10. Study Endpoints

The endpoints of the study were the freedom from episodes of AF, defined as the absence of episodes of AF lasting >30 seconds on any ECG or 24-hour ECG Holter monitoring [2], and the identification of cytokines and SERCA levels comparing patients with sinus rhythm restoration (responders) vs. patients with AF recurrence (nonresponders) after epicardial ablation at 12-month follow-up. 

### 2.11. Follow-Up

At outpatient clinic, patients were followed for 10 days after the epicardial ablation procedure to evaluate AF recurrence. In case of AF recurrence during the first three months, patients received a pharmacological and/or an external cardioversion to abolish AF, and to restore sinus rhythm. After a blanking period, we discontinued AADs medications, as recommended by international guidelines [2]. Six months after epicardial ablation we discontinued oral anticoagulation in case of CHADS_2_ score <1 [2]. We detected AF recurrence during the clinical evaluation at outpatient clinic by symptoms, ECG registration, ECG Holter exams, and hospital discharge schedules.

### 2.12. Statistical Analysis

All data were analyzed by two different physicians, and the patients (n = 27) were divided after ablation in responder patients (n = 15) vs. nonresponders (n = 12) to the ablative treatment. Based on our pilot studies and on the prevalence of persistent AF treated via thoracoscopic epicardial AF ablation, we performed a power analysis to determine the optimal sample size: Applying an α cut-off of 5% and a β cut-off of 20%, we calculated that n = 12 per group would have been sufficient to reach statistical significance. We applied the two-tailed Student’s t test to test normally distributed variables, for paired or unpaired data. We used one-way analysis of variance (ANOVA) for more than two independent groups of data. Chi-square or Fisher exact test was used to compare categorical variables. We defined as statistical significant a *p* value <0.05. Overall survival and event-free survival were assessed by Kaplan–Meier survival curves and compared applying the log-rank test, dividing the overall study population into three groups (tertiles) according to SERCA values. We performed a multivariable logistic regression analysis for AF recurrence event risk. Only variables presenting a *p* value ≤0.25 at the univariate analysis were included in the model. We used a stepwise method with backward elimination, and we calculated odds ratios (OR) with 95% confidence intervals. The model was evaluated with a Hosmer and Lemeshow test. Statistical analysis was performed using the SPSS software (SPSS Inc, Chicago, IL, USA). 

## 3. Results

**Figure 1:**
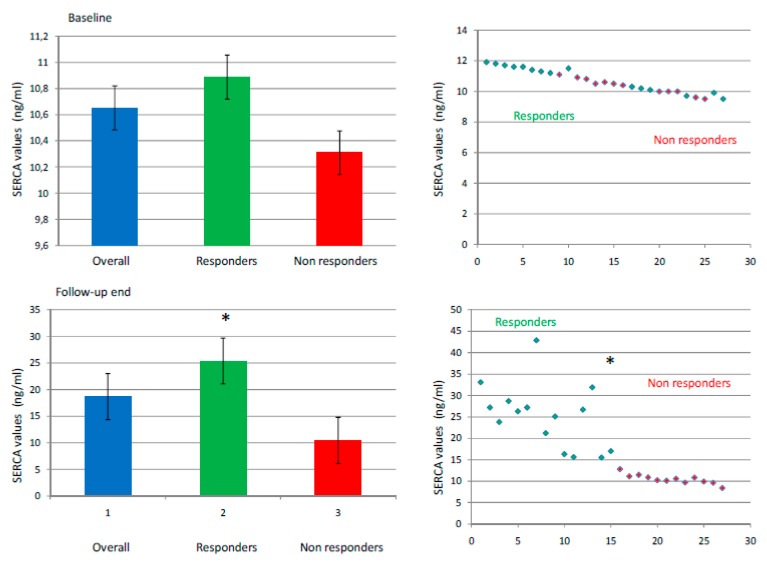
Baseline (top) and follow-up end (bottom) levels of sarcoplasmic endoplasmic reticulum calcium ATPase (SERCA) in nanograms/milliliters (ng/mL). In the left part of the figure, the SERCA levels are represented by columns with mean values ± standard deviations in overall population (blue), and responders (green color) vs. nonresponders (red) patients. On the right part of the figure, the dispersion graph represents the values of SERCA in responders (green) vs. nonresponders (red color). The symbol “*” marks a *p* value < 0.05 as statistically significant.

**Figure 2:**
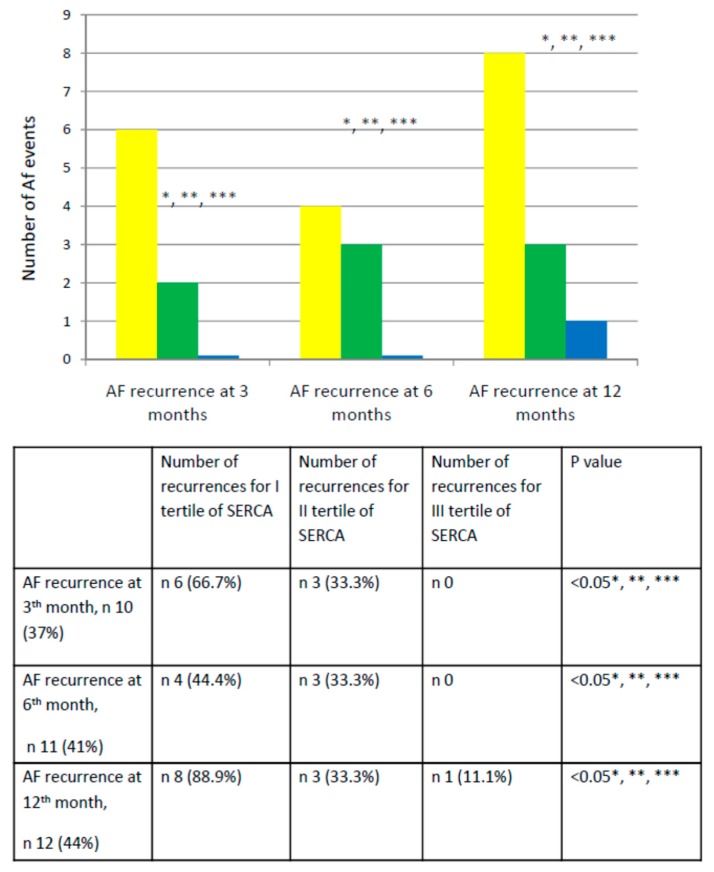
In the upper part of the figure, the number of atrial fibrillation (AF) recurrence for 3, 6, and 12 months of follow-up in study population divided for tertiles of sarcoplasmic endoplasmic reticulum calcium ATPase (SERCA) values and arranged by columns for number of events in ascending order (in yellow the I tertile of SERCA, in green the II tertile, and in blue the III tertile). In the lower part of figure, the table to report the number (n) of AF recurrences for I, II, and III tertile of SERCA values. The symbol “*” is for *p* <0.05, comparing I tertile vs. II tertile of SERCA; the symbol “**” is for *p* < 0.05, comparing I vs. III tertile of SERCA; the symbol “***” is for *p* < 0.05, comparing II vs. III tertile of SERCA.

**Figure 3:**
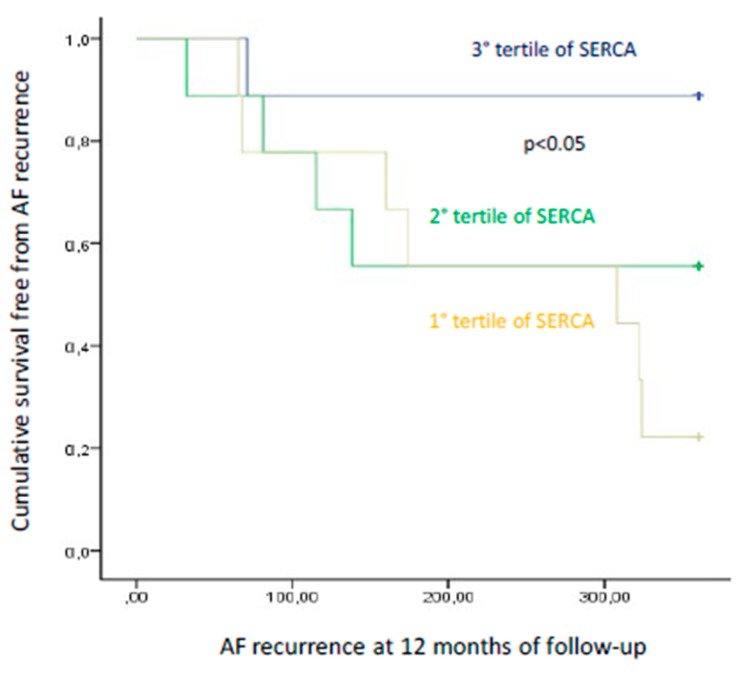
Kaplan curve for cumulative survival free from atrial fibrillation (AF) events dividing study population into tertiles of sarcoplasmic endoplasmic reticulum calcium ATPase (SERCA) values. Yellow: 1° tertile; green: 2° tertile; blue: 3° tertile. We used a log-rank test for the equality of survival distributions for the different levels and tertiles of SERCA, with Χ^2^ = 6.241 and *p* < 0.05 considered as statistically significant.

We recruited 27 patients (27% female); mean age was 57.1 ± 5.8 years; mean body mass index (BMI) was 28.2 ± 2 (kg/m^2^). We reported the clinical characteristics of our study population in Table 1. At baseline, these characteristics were well balanced comparing responders vs. nonresponders to the ablative treatment, except the mean AF duration time in months (40.2 ± 7.5 vs. 50.2 ± 5.7, *p* < 0.05), left atrium (LA) diameter (LAD, in millimeters, mm: 42.2 ± 3.9 vs. 47.8 ± 4.0, *p* < 0.05), and LA volume (LAV, in milliliters, mL: 30.5 ± 2.5 vs. 36.2 ± 2.5, *p* < 0.05). At the follow-up end, the responders to epicardial ablation showed a significant reduction of inflammatory biomarkers, BNP, and significantly augmented levels of SERCA compared to nonresponders (*p* < 0.05). Equally important, responders also exhibited lower LAD (41.2 ± 2.9 vs. 48.2 ± 4.3 mm, p < 0.05), LAV (28.5 ± 2.3 vs. 36.8 ± 2.9 mL, *p* < 0.05), and a significantly improved myocardial function, assessed in terms of left ventricle ejection fraction (LVEF, 52 + 4 vs. 46 + 4%, *p* < 0.05), as well as a reduced mitral regurgitation (MR) compared to nonresponder patients. The different expressions of SERCA at baseline and at follow-up in responders vs. nonresponders to epicardial ablation is depicted in Figure 1. Dividing the overall study population to stratify the risk of AF events into three groups (tertiles: I tertile ≤ 9.18 ng/mL; 9.18 ≤ II tertile ≤ 11.21 ng/mL; III tertile ≥ 11.21 mg/dL) according to SERCA values, we observed a significantly different rate of AF recurrence at the third, sixth, and 12th month of follow-up comparing patients in I vs. II and vs. III tertile of SERCA (*p* < 0.05). Figure 2 represents the Kaplan curve, and log-rank tests compared AF recurrent event rates across the tertiles of baseline SERCA values (patients in I vs. II vs. III tertile of SERCA, (χ^2^ = 6.241, *p* < 0.05)). At the end of follow-up, responders to epicardial ablation were taking fewer AADs, VKA, and new oral anticoagulants drugs vs. nonresponders (*p* < 0.05) (Figure 3). At the multivariate analysis, we tested by Cox regression the factors influencing AF recurrences at one-year follow-up after thoracoscopic epicardial AF ablation (confidence interval (CI) 95%, *p* < 0.05) (Table 1). As shown in Table 2, these factors were: Mean AF duration (HR 1.235 (1.037–1.471), *p* < 0.05), LAV (HR 1.755 (1.126–2.738), *p* < 0.05), BNP (HR 1.945 (1.895–1.999), *p* < 0.05), and SERCA (HR 1.763 (1.167–2.663), *p* < 0.05).

## 4. Discussion

The main findings of the present study are: (1) Responders to epicardial ablation showed a significant reduction of BNP values and inflammatory cytokines vs. nonresponders; (2) responders displayed a significant reduction of LAD, LAV, and MR and a significant improvement of LVEF vs. nonresponders; (3) responders had significantly increased SERCA levels in peripheral lymphocytes vs. nonresponders; and (4) AF recurrence after epicardial ablation was predicted via AF duration, LAV, SERCA levels, and serum BNP.

BNP is a cardiac peptide implied in the pathogenesis and persistence of AF [18,19]. In isolated rabbit cardiomyocytes, BNP infusion increases transient inward Ca^2+^ currents, sodium (Na^+^) and Na^+^/Ca^2+^ exchanger currents, and L-type Ca^2+^currents [19]. Such alterations in Ca^2+^ currents increase arrhythmogenesis, amplifying genesis and persistence of AF [10,19]. In fact, increased BNP levels after an ablative approach in AF patients associate with a greater risk of future AF recurrence [20], and could be predictive of AF recurrence after epicardial ablation. Similarly, the inflammation is considered a key contributor to the pathophysiology of AF, also in patients who do not respond to an ablative approach [21]. In our study, nonresponders had higher values of LAD and LAV both at baseline and at follow-up. Increased values of LAD and LAV are established markers of atrial remodeling [19,20,21]. Atrial remodeling is crucial in the pathogenesis of AF [22] and might underlie the failure of an ablative treatment [23]. Indeed, atrial remodeling might cause AF resistance and predispose the patients with persistent AF to higher risk of AF recurrence after an ablative approach [19,20,21,22,23,24,25]. On the other hand, responders to ablation might experience an amelioration in the volumetry of cardiac chambers, with consequent improvement of LVEF [26], which, alongside the significant reduction in MR observed in responders, might be interpreted as an index of mechanical improvement (amelioration in diastolic and systolic cardiac phases) induced by successful epicardial ablation [27]. AF has been shown to aggravate MR by AF-induced atrial remodeling [27,28]. Indeed, AF can alter atrial function and synchrony, affecting annular size, geometry, and function [28]. These abnormalities can favor AF onset, recurrence, and persistence [27,28,29]. Consequently, patients with long-standing AF might experience a lower rate of sinus rhythm restoration after an epicardial ablative approach [30]. Hence, AF duration can affect the outcomes of catheter ablation treatment [30]. AF persistence might contribute to alterations in the electrophysiological and anatomical properties of the heart, becoming itself a cause, as well as a resulting effect of the electro-anatomical atrial remodeling, which is a fundamental determinant of AF perpetuation [7,30]. These complex electro-anatomical modifications have been shown to make patients more refractory to ablative therapies [16,22]. Notably, after sinus rhythm restoration at one-year follow-up, responders exhibited significantly higher levels of SERCA values compared to baseline, as well as compared to patients nonresponding to epicardial ablation (*p* < 0.05). SERCA is a protein implied in the handling of intracellular Ca^2+^ [12], and sinus rhythm restoration induced by epicardial ablation in responder vs. nonresponder patients could trigger an over activation/expression of SERCA. Consequently, this effect could reduce Ca^2+^ overload in human atrial myocytes, then leading to reduction of DADs and atrial arrhythmic events [12]. This ameliorative effect on Ca^2+^ handling could reduce AF persistence and atrial remodeling [14,15,16]. Indeed, we reported a significantly different number of AF recurrences at the third, sixth, and 12^th^ month of follow-up comparing patients in I vs. II vs. III tertile of SERCA (*p* < 0.05). Moreover, patients with lower expression of SERCA had a higher rate of AF recurrence for all follow-up duration. This result has been shown in the bar graphs as well as in the Kaplan curves that compared AF recurrent event rates across the tertiles of baseline SERCA values (χ^2^ = 6.241, *p* < 0.05). Therefore, patients in the III tertile of SERCA exhibit higher and significant cumulative survival free from AF recurrences; henceforth, lowest values of SERCA could mark patients with persistent AF more refractory to ablative therapies [16,22]. Indeed, a lower baseline SERCA expression in patients with persistent AF might increase ~1.8 fold the risk to experience AF recurrences after epicardial ablation. Taken together, our findings indicate that the analysis of SERCA levels by peripheral blood lymphocytes assay could give precious information on pro-arrhythmogenesis and on the response to epicardial ablation for patients with persistent AF. This aspect might help clinicians to identify patients who may not respond to epicardial ablation. Additionally, SERCA may become a therapeutic target of tailored therapies and interventional approaches to reduce the arrhythmic burden and recurrences of patients with persistent AF and treated by an ablative approach.

### Study Limitations

One limitation of our prospective multicenter study is the small size of our population of patients with persistent AF treated by epicardial catheter ablation. However, we performed a priori power analysis revealing that n = 12 per group would have been sufficient to achieve statistical significance. Unfortunately, the small size did not allow building a robust predictive model. Moreover, due to our relatively short follow-up (12 months), we cannot draw conclusions for long-term prognosis. In this study, we did not systematically use continuous ECG monitoring systems to evaluate AF recurrence, and/or remote monitoring systems, which have been previously reported to affect prognosis [30]. We did not report data on cardiac imaging via magnetic resonance to assess cardiac fibrosis. Finally, the low percentage of responders to an epicardial ablative treatment might be due to the fact that we did not perform a combined epicardial and endocardial approach to reduce AF recurrences, as described by other investigators [6].

## 5. Conclusions

Our data indicate that, at baseline, responders to AF ablation displayed an expression of SERCA that was not different compared with nonresponders. After a successful epicardial ablation procedure, there was significant difference of SERCA expression comparing responders vs. nonresponders. However, this finding could be secondary to sinus rhythm restoration induced by successful epicardial ablation. Additionally, responders to epicardial ablation exhibited a reduction in BNP and inflammatory markers. They also showed a marked reduction in atrium size and MR, with overall improved LVEF. Taken together, our findings indicate that targeting SERCA may represent an effective therapeutic strategy to reduce post-ablative recurrences in patients with persistent AF.

## Figures and Tables

**Table 1:** Clinical characteristics of the study population before treatment (baseline) and at follow-up.

		At Baseline			At Follow-up End		
Clinical General Characteristics	General Population	Responders	Non Responders	P value	Responders	Non Responders	P value
Number of patients	n 27	n 15 (56%)	n 12 (44%)		n 15 (56%)	n 12 (44%)	
Age (years)	57.1 ± 5.8	55.6 ± 5.8	59 ± 5.3	--	/	/	--
Gender (male)	n 17 (63%)	n 9 (60%)	n 8 (66%)	--	/	/	--
BMI (kg/m^2^)	28.2 ± 2	27.7 ± 1.8	28.9 ± 2.1	--	27.9 ± 1.7	28.4 ± 1.9	--
Diabetes	n 4 (15%)	n 2 (13%)	n 2 (16%)	--	n 2 (13%)	n 2 (16%)	--
CAD	n 10 (37%)	n 6 (40%)	n 4 (34%)	--	n 6 (40%)	n 5 (41.7%)	--
COPD	n 5 (18.5%)	n 3 (20%)	n 2 (17%)	--	n 3 (20%)	n 2 (17%)	--
Hypertension	n 6 (22%)	n 3 (20%)	n 3 (25%)	--	n 4 (26.7%)	n 3 (25%)	--
History of AF duration (months)	44.7 ± 8.3	40.2 ± 7.5	50.2 ± 5.7	<0.05*	/	/	--
Previous stroke	n 2 (0.7%)	n 1 (0.6%)	n 1 (0.8%)	--	/	/	--
**Biohumoral markers**							
Creatinine (mg/dL)	0.98 ± 0.18	1.0 ± 0.17	0.96 ± 0.20	--	0.98 ± 0.21	1.01 ± 0.19	--
BNP (pg/mL)	198.59 ± 9.6	257.54 ± 6.12	287. 15 ± 56.26	--	43.67 ± 4.97	303.75 ± 51.16	<0.05**
SERCA (ng/mL)	10.6 ± 0.8	10.9 ± 0.8	10.3 ± 0.6	--	25.4 ± 6.2	10.6 ± 0.9	<0.05**
IL-6 (pg/mL)	2.8 ± 0.8	2.7 ± 0.9	2.9 ± 0.7	--	1.6 ± 0.2	2.4 ± 0.3	<0.05**
TNFα (pg/mL)	9.1 ± 2.3	9.3 ± 2.1	8.6 ± 2.6	--	6.1 ± 1.7	8.7 ± 2.8	<0.05**
CRP (mg/dl)	4.0 ± 0.2	4.1 ± 0.2	3.9 ± 0.3	--	1.9 ± 0.4	3.3 ± 0.6	<0.05**
**Echocardiographic measurements**							
LAD* (mm)	44.15 ± 5.1	42.2 ± 3.9	47.8 ± 4.0	<0.05*	41.2 ± 2.9	48.2 ± 4.3	<0.05**
LAV* (ml)	33 ± 3.8	30.5 ± 2.5	36.2 ± 2.5	<0.05*	28.5 ± 2.9	36.8 ± 2.9	<0.05**
LVEF	49 ± 5	51 ± 4	50 ± 2	--	52 ± 4	46 ± 4	<0.05**
MR low grade	n 19 (70%)	n 10 (66.7%)	n 8 (67%)	--	n 13 (86%)	n 7 (58.3%)	<0.05**
MR moderate grade	n 9 (33%)	n 5 (33%)	n 4 (33%)	--	n 2 (13.3%)	n 5 (41.7%)	<0.05**
**Drug Therapy**							
Beta blockers	n 6 (22.2%)	n 3 (20%)	n 3 (25%)	--	n 2 (13.3%)	n 2 (16.6%)	--
ACE inhibitors	n 2 (7.4%)	n 1(5%)	n 1 (8%)	--	n 1 (5%)	n 1 (8%)	--
ARS inhibitors	n 4 (15%)	n 2 (13%)	n 2 (16%)	--	n 2 (13%)	n 2 (16%)	--
AADs class 1	n 5 (18%)	n 3 (20%)	n 2 (17%)	--	n 2 (13%)	n 3 (25%)	<0.05**
AADs class 3	n 15 (56%)	n 8 (53%)	n 7 (58%)	--	n 2 (13%)	n 7 (67%)	<0.05**
Vitamin K Antagonists	n 11 (41%)	n 6 (40%)	n 5 (42%)	--	n 3 (20%)	n 6 (50%)	<0.05**
New oral anticoagulation	n 16 (59%)	n 9 (60%)	n 7 (58%)	--	n 4 (27%)	n 6 (50%)	<0.05**

The patients are divided in general population, and differentiated in responders vs. nonresponders to epicardial ablation. AAD is antiarrhythmic drugs; ACE is angiotensin converting enzyme; AF is atrial fibrillation; ARS is angiotensin receptors; BMI is body mass index; BNP is B type natriuretic peptide; CAD is coronary artery disease; COPD is chronic obstructive pulmonary disease; CRP is c reactive protein; IL-6 is interleukin 6; LAD is left atrium diameter; LAV is left atrium volume; LVEF is left ventricle ejection fraction; MR is mitral regurgitation; SERCA is sarcoplasmic endoplasmic reticulum calcium ATPase; TNFα is tumor necrosis factor α. The symbol “--“ is indicating a nonsignificant p value comparing responders vs. nonresponders (*p* value >0.05); the symbol “*” is indicating a *p* value < 0.05 at baseline comparing responders vs. nonresponders; the symbol “**” is indicating a *p* value < 0.05 at follow-up end comparing responders vs. nonresponders.

**Table 2:** Univariate and multivariate analysis for atrial fibrillation (AF) recurrence after epicardial ablation at 12 months of follow-up.

		Univariate analysis			Multivariate analysis	
Variable	OR	CI 95%	P value	OR	CI 95%	P value
Diabetes	0.933	(0.204–4.274)	0.929	0.338	(0.025–4.637)	0.417
Obesity	1.312	(0.950–1.811)	0.101	1.473	(0.841–2.580)	0.175
Age	1.082	(0.956–1.225)	0.212	0.805	(0.637–1.018)	0.070
Mean AF duration	1.121	(1.032–1.218)	0.007	1.235	(1.037–1.471)	0.018*
LVEF	0.918	(0.817–1.032)	0.153	0.746	(0.555–1.003)	0.053
LA volume	1.264	(1.038–1.540)	0.020	1.755	(1.126–2.738)	0.013*
BNP	1.021	(1.005–1.037)	0.001	1.945	(1.895–1.999)	0.045*
SERCA	1.221	(1.105–1.349)	0.001	1.763	(1.167–2.663)	0.007*

A p value < 0.05 was considered significant and marked with the symbol *. AF is atrial fibrillation; BNP is B type natriuretic peptide; LA is left atrium; LVEF is left ventricle ejection fraction; SERCA is sarcoplasmic endoplasmic reticulum calcium ATPase. CI is interval of confidence; OR is odds ratio.
